# Degranulation of mast cells induced by gastric cancer-derived adrenomedullin prompts gastric cancer progression

**DOI:** 10.1038/s41419-018-1100-1

**Published:** 2018-10-10

**Authors:** Yi-pin Lv, Liu-sheng Peng, Qi-hong Wang, Na Chen, Yong-sheng Teng, Ting-ting Wang, Fang-yuan Mao, Jin-yu Zhang, Ping Cheng, Yu-gang Liu, Hui Kong, Xiao-long Wu, Chuan-jie Hao, Weisan Chen, Jiang Zhu, Bin Han, Qiang Ma, Ke Li, Quanming Zou, Yuan Zhuang

**Affiliations:** 1 National Engineering Research Centre of Immunological Products, Department of Microbiology and Biochemical Pharmacy, College of Pharmacy, Third Military Medical University, Chongqing, China; 2 La Trobe Institute of Molecular Science, School of Molecular Science, La Trobe University, Bundoora, VIC 3085 Australia; 3 Institute of Pathology and Southwest Cancer Center, Southwest Hospital, Third Military Medical University, and Key Laboratory of Tumor Immunopathology, Chongqing, 400038 China; 4 Department of Pharmacy, Affiliated Hospital of North Sichuan Medical College, Nanchong, Sichuan Province China; 5 Department of General Surgery, Chongqing Hospital of Traditional Chinese Medicine, Chongqing, China

## Abstract

Mast cells are prominent components of solid tumors and exhibit distinct phenotypes in different tumor microenvironments. However, their precise mechanism of communication in gastric cancer remains largely unclear. Here, we found that patients with GC showed a significantly higher mast cell infiltration in tumors. Mast cell levels increased with tumor progression and independently predicted reduced overall survival. Tumor-derived adrenomedullin (ADM) induced mast cell degranulation via PI3K-AKT signaling pathway, which effectively promoted the proliferation and inhibited the apoptosis of GC cells in vitro and contributed to the growth and progression of GC tumors in vivo, and the effect could be reversed by blocking interleukin (IL)-17A production from these mast cells. Our results illuminate a novel protumorigenic role and associated mechanism of mast cells in GC, and also provide functional evidence for these mast cells to prevent, and to treat this immunopathogenesis feature of GC.

## Introduction

Gastric cancer (GC) is a severe health problem, being the fourth most common malignancies and the second leading cause of cancer death worldwide^1^. Despite significant advances in prevention, diagnose, and therapeutic options and strategies in these years, many unanswered questions remain, particularly the pathogenesis of GC is not elaborated clearly. Nowadays, It is generally accepted that the development and prognosis of GC is influenced by tumor and host immune system cross-talk^2,3^, with some studies supporting a crucial role for adaptive immunity in determining the clinical outcomes of GC patients^4–6^. However, the role of innate immunity and innate immune cell is little known during GC progression.

Mast cells are a group of innate immune cells with profound immune-regulatory effects on tumor progression^7^, such as angiogenesis^8^, interaction with other immune cells and remodeling tumor microenvironment^9,10^. Currently, some researches performed on mast cells in GC and these limited studies are mostly focused on the correlation between GC survival rate and their mast cell infiltration by immunohistochemistry^11^, and some on the relationships between the density of the infiltrating mast cells and local angiogenesis^12,13^. Overall, these studies suggested that mast cells might be promising therapeutic targets for GC. However, the presence of tumor-associated mast cells, as well as their precise mechanisms of communication in gastric cancer remains largely unclear.

Adrenomedullin (ADM) is a 52-amino acid peptide amide, which had been discovered from a human pheochromocytoma^14^. It plays a powerful role in human carcinogenesis through diverse mechanisms^15^. Recent studies has shown that elevated ADM expression in cancer cells can augment angiogenesis, reduce apoptosis, and even promote tumor proliferation^16,17^. In addition to its known tumorigenic abilities, ADM has been shown to regulate certain aspects of the immune function that include modulating mast cell activation^18^, which potentially involved with tumor promotion and progression.

Herein, we investigated the interplays among mast cells, ADM and tumor cells in the GC microenvironment. We show that mast cells are highly infiltrated in GC, and tumor-derived ADM activates mast cell degranulation via PI3K-AKT signaling pathway. In turn, activated mast cells release interleukin (IL)-17A, which can promote tumor cell proliferation and suppress its apoptosis in vitro. Besides, blocking mast cells degranulation and associated-IL-17A can inhibit tumor growth and GC progression in vivo. Our data confirm a protumorigenic role of mast cells in GC. These tumor-infiltrating mast cells increase with tumor progression and are negatively correlated with patient survival after surgery, suggesting that mast cells may be a novel target to improve GC therapy.

## Results

### Mast cells are enriched in GC as tumor progress and independently predict poor patient survival

**Fig. 1:**
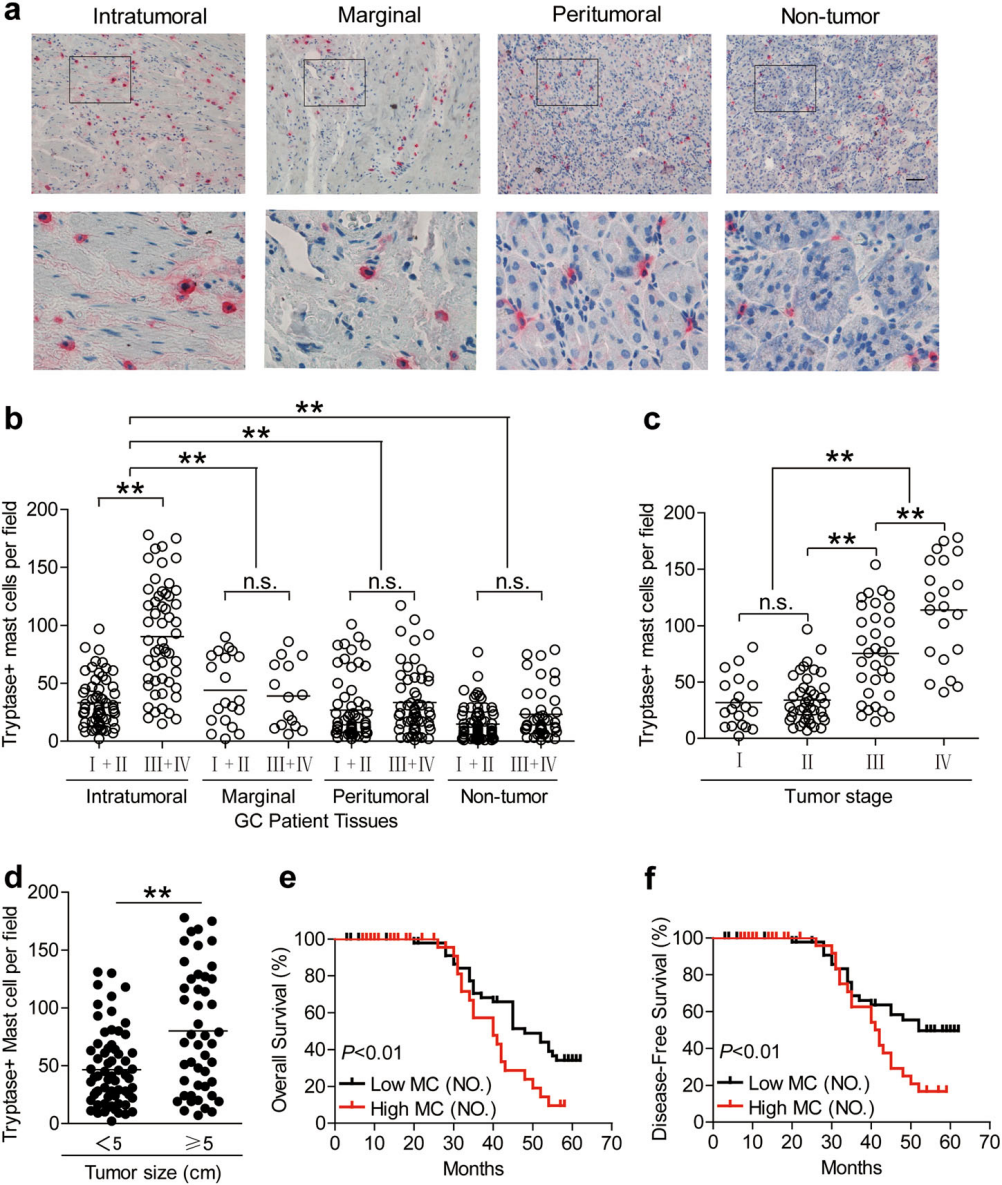
Mast cells accumulate in GC tumors with disease progression and predict poor patient survival. **a** Representative analysis of tryptase^+^ (red) mast cell distributions in intratumoral, marginal, peritumoral, and non-tumor tissues of GC patients by immunohistochemical staining. Scale bars: 100 microns. **b** The total number of mast cells per field among TNM stages (I + II vs III + IV) in each tissue of patients with GC by immunohistochemical staining. Cumulative results from 114 GC patients were shown. **c** Intratumoral mast cell per field among TNM stages was compared. **d** Intratumoral mast cell per field were correlated with increased tumor size. **e**, **f** Kaplan–Meier plots for overall survival and disease-free survival by median mast cell per field (median level: 47/per field).The horizontal bars in panels **b**–**d** represent mean values. Each ring/dot in panels **b**–**d** represents 1 patient. **P < 0.01, n.s., *P* > 0.05 for groups connected by horizontal lines. MC (NO.), mast cell per field number

To evaluate the potential role of mast cells in human GC, we analyzed the infiltration of mast cell from intratumoral, marginal, peritumoral, and non-tumor tissues of GC patients at various stages by Immunohistochemistry. Notably, patients with GC showed a higher mast cell infiltration in intratumoral tissues than marginal, peritumoral, and non-tumor tissues (Fig. 1a). Moreover, as the cancer progressed, the accumulation of intratumoral mast cells increased significantly (Fig. 1b). This intratumoral mast cell accumulation was most notable from stage II onwards (Fig. 1c), indicating a potential role for mast cells in the GC microenvironment. In keeping with this finding, increased mast cell per field was correlated with increased advanced tumor size (Fig. 1d).

Next, we evaluated the clinical relevance of intratumoral mast cells in GC. Comparing patients with high (≥47/per field median level) versus low (<47/ per field) mast cell per field number, the 62-month overall survival rates were significantly lower for those within the higher per field number (Fig. 1e). And the disease-free survival rates were similar to overall survival rates (Fig. 1f). Taken together, these findings suggest that increased intratumoral mast cells are associated with tumor progression and poor survival of GC patients.

### TTCS can stimulate mast cell activation and degranulation via PI3K-AKT signaling pathway

**Fig. 2:**
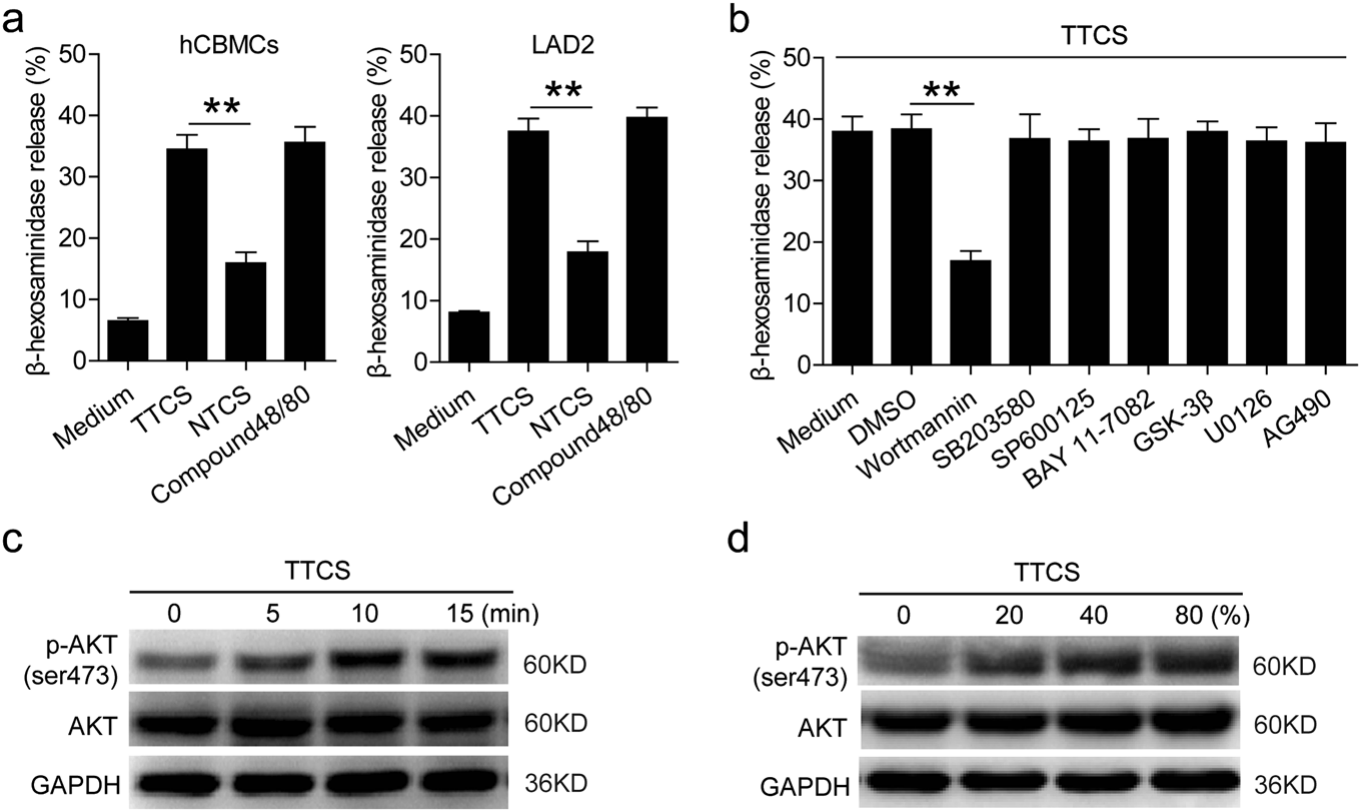
TTCS induces mast cell activation and degranulation via PI3K-AKT signaling pathway. **a** The degranulation of hCBMCs and LAD2 cells stimulated by TTCS or NTCS was analyzed (*n* = 3). **b** The degranulation of LAD2 cells exposed to TTCS with or without signal transduction inhibitors was analyzed (*n* = 5). **c**, **d** AKT and p-AKT in LAD2 cells exposed to TTCS (5, 10, 15 min) or exposed to TTCS with different concentrations (20%, 40%, 80%) were analyzed by western blot. *P < 0.05, **P < 0.01 for groups connected by horizontal lines. hCBMCs, human umbilical cord blood-derived cultured mast cells

Degranulation is an important biological activity for mast cells. To see whether the tumor microenvironment might play an important role in this process, we stimulated mast cells with TTCS, and found that, compared to NTCS, TTCS significantly induced mast cell degranulation (Fig. 2a). As a positive control for stimulating mast cell degranulation, Compound 48/80 appeared here. To reveal which signaling pathways might operate in the degranulation of mast cells, we first pretreated mast cells with corresponding inhibitors including AG490 (a JAK inhibitor), BAY 11-7082 (an IκBα inhibitor), SP600125 (a JNK inhibitor), SB203580 (a MAPK inhibitor), U0126 (MEK-1 and MEK-2 inhibitor), or Wortmannin (a PI3K inhibitor), and then exposed them to the indicated TTCS. The results showed that only blocking the signal transduction of PI3K with Wortmannin effectively suppressed the degranulation of mast cells (Fig. 2b). Furthermore, AKT, direct PI3K-AKT pathway downstream substrate, was predominantly phosphorylated in mast cells after treatment with TTCS in a time-dependent manner (Fig. 2c). Interestingly, with the increased concentration of TTCS, the phosphorylation level of AKT was up-regulated in mast cells (Fig. 2d). These results above indicate that, in the GC microenvironment, mast cells can be activated and lead to degranulation via PI3K-AKT signaling pathway.

### The degranulation of mast cell can be regulated by tumor-derived factor ADM in GC

**Fig. 3:**
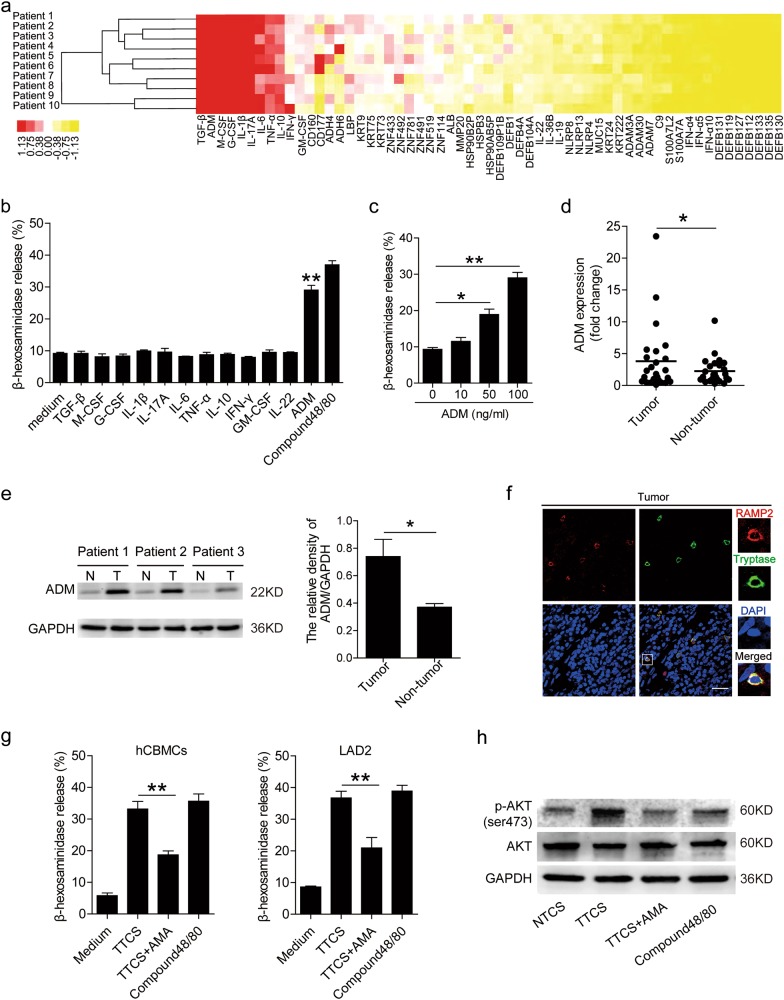
Tumor-derived factor ADM stimulates mast cell degranulation. **a** Clustering of microarray data for the expression of 60 pro-inflammatory molecule genes in human tumor tissues from 10 GC patients. **b** The degranulation of LAD2 cells exposed to GM-CSF, M-CSF, G-CSF, IFN-γ, ADM, TGF-β, TNF-α, IL-1β, IL-6, IL-10, IL-17A, IL-22 (100 ng/ml) was analyzed (*n* = 3). **c** The degranulation of LAD2 cells stimulated by ADM was analyzed (*n* = 3). **d** ADM expression between autologous tumor and non-tumor tissues (*n* = 25) was analyzed by real-time PCR. **e** ADM production between autologous tumor (T) and non-tumor (N) tissues (3 pairs) were analyzed by western blot. **f** Representative analysis of receptor activity modifying protein 2 (RAMP2) (specific subunit of ADM receptor 1 (ADMR1) heterodimer)-expressing (red) tryptase^+^ mast cells (green) in tumor tissues of GC patients by immunofluorescence. Scale bars: 50 microns. **g** The degranulation of mast cells exposed to TTCS with or without AMA was analyzed (*n* = 5). **h** AKT and p-AKT in LAD2 cells exposed to TTCS with or without AMA (10 min) were analyzed by western blot. The horizontal bars in panel (**d**) represent mean values. *P < 0.05, **P < 0.01 for groups connected by horizontal lines. hCBMCs human umbilical cord blood-derived cultured mast cells, GC gastric cancer

Tumor microenvironment can possess various soluble factors, including pro-inflammatory molecules. To see which molecules might have effects on mast cells, we first screened pro-inflammatory molecules in human GC microenvironments by microarray (Fig. 3a), and stimulated mast cells with highly-expressed molecules including GM-CSF, M-CSF, G-CSF, IFN-γ, ADM, TGF-β, TNF-α, IL-1β, IL-6, IL-10, IL-17A, IL-22, etc. We found that only ADM remarkably induced mast cell degranulation in a dose-dependent manner (Fig. 3b, c). Next, we found that ADM in tumor tissues was significantly increased when compared to that in non-tumor tissues (Fig. 3d, e), and that a positive correlation between mast cell degranulation (Supplementary Figure 2a) and ADM production most likely derived from EpCam^+^ tumor cells within GC tumors (Supplementary Figure 2b). Furthermore, the expression of ADM in TTCS was significantly elevated when compared to that in NTCS (Supplementary Figure 2c). To evaluate the potential role of GC tumor-derived ADM in the degranulation of mast cells which expressed RAMP2 (specific subunit of ADM receptor 1 heterodimer) within tumors (Fig. 3f), we added ADMR antagonist AMA into TTCS/mast cell co-culture system. Interestingly, blockade of ADM-ADMR interaction efficiently inhibited mast cell degranulation (Fig. 3g). Consistent with these findings, AKT phosphorylation was abolished when blocking tumor derived-ADM (Fig. 3h). These findings show that tumor-derived ADM plays an essential role in mast cell degranulation by activation of PI3K-AKT signaling pathway in the GC microenvironment.

### Blockade of mast cell-associated degranulation inhibits tumor growth and GC progression

**Fig. 4:**
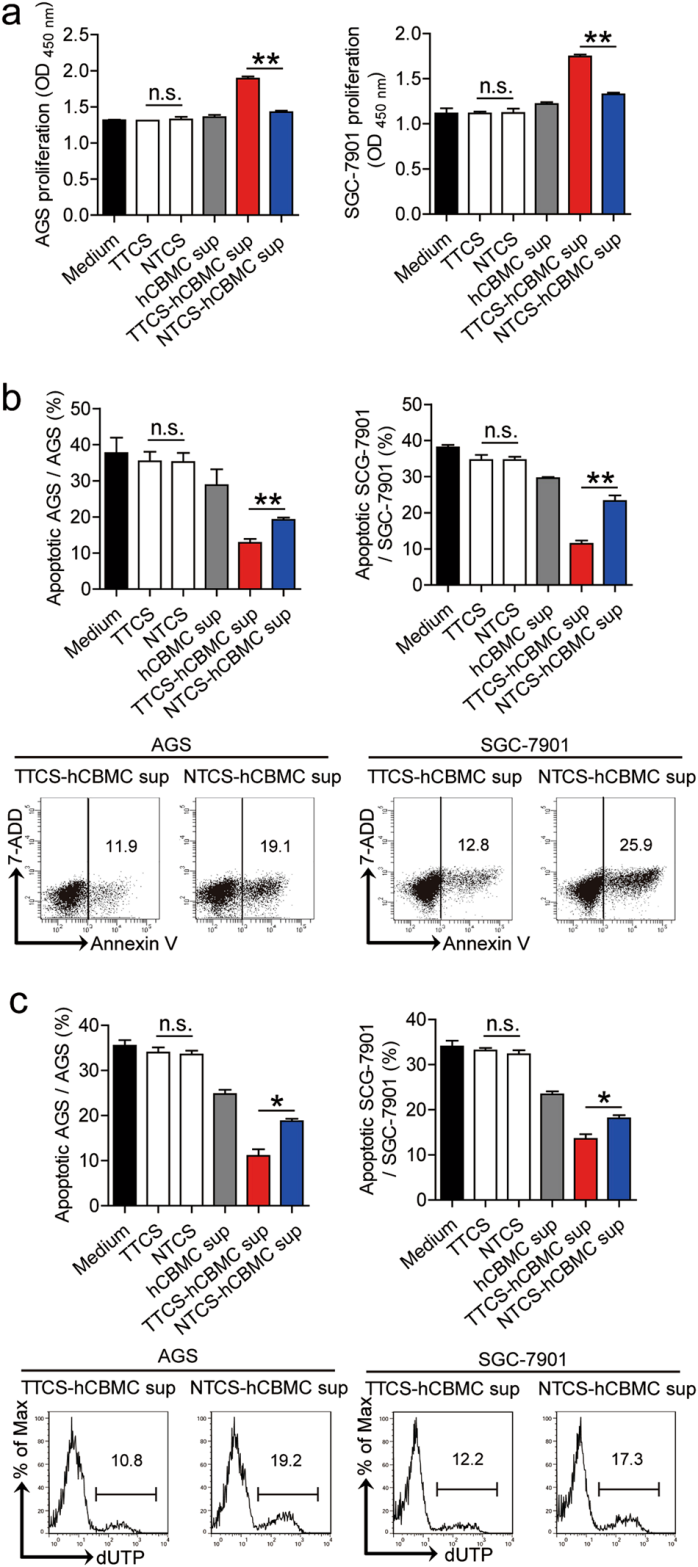
Mast cell-associated degranulation promotes gastric cancer cells proliferation and inhibits them apoptosis in vitro. **a**–**c** GC cells were stimulated with different culture supernatants, as described in Methods. The proliferation **a** of GC cells were analyzed (*n* = 3). The apoptosis of GC cells were analyzed by annexin V (**b**) and deoxyuridine triphosphate nucleotides (dUTP) (**c**) detection (*n* = 3). *P < 0.05, **P < 0.01, n.s., *P* > 0.05 for groups connected by horizontal lines

To test the effect of mast cell-associated degranulation on GC cells, we stimulated GC cells with the culture supernatants from TTCS-conditioned hCBMCs (referred as TTCS-hCBMCs in the following text). This potently induced GC cell proliferation compared to the culture supernatants from NTCS-conditioned hCBMCs (referred as NTCS-hCBMCs in the following text) (Fig. 4a). We also found that, compared to those exposed to the culture supernatants from NTCS-hCBMCs, GC cells exposed to the culture supernatants from TTCS-hCBMCs exhibited a delayed onset of apoptosis assessed by annexin V (Fig. 4b) and deoxyuridine triphosphate nucleotides (dUTP) (Fig. 4c) detection. Similar observations were made when using the culture supernatants from TTCS-conditioned LAD2 cells (referred as TTCS-LAD2 in the following text)) (Supplementary Figure 3a–c). Together, these results suggest that mast cell-associated degranulation-derived culture supernatants promote the proliferation and inhibit the apoptosis of GC cells in vitro.

**Fig. 5:**
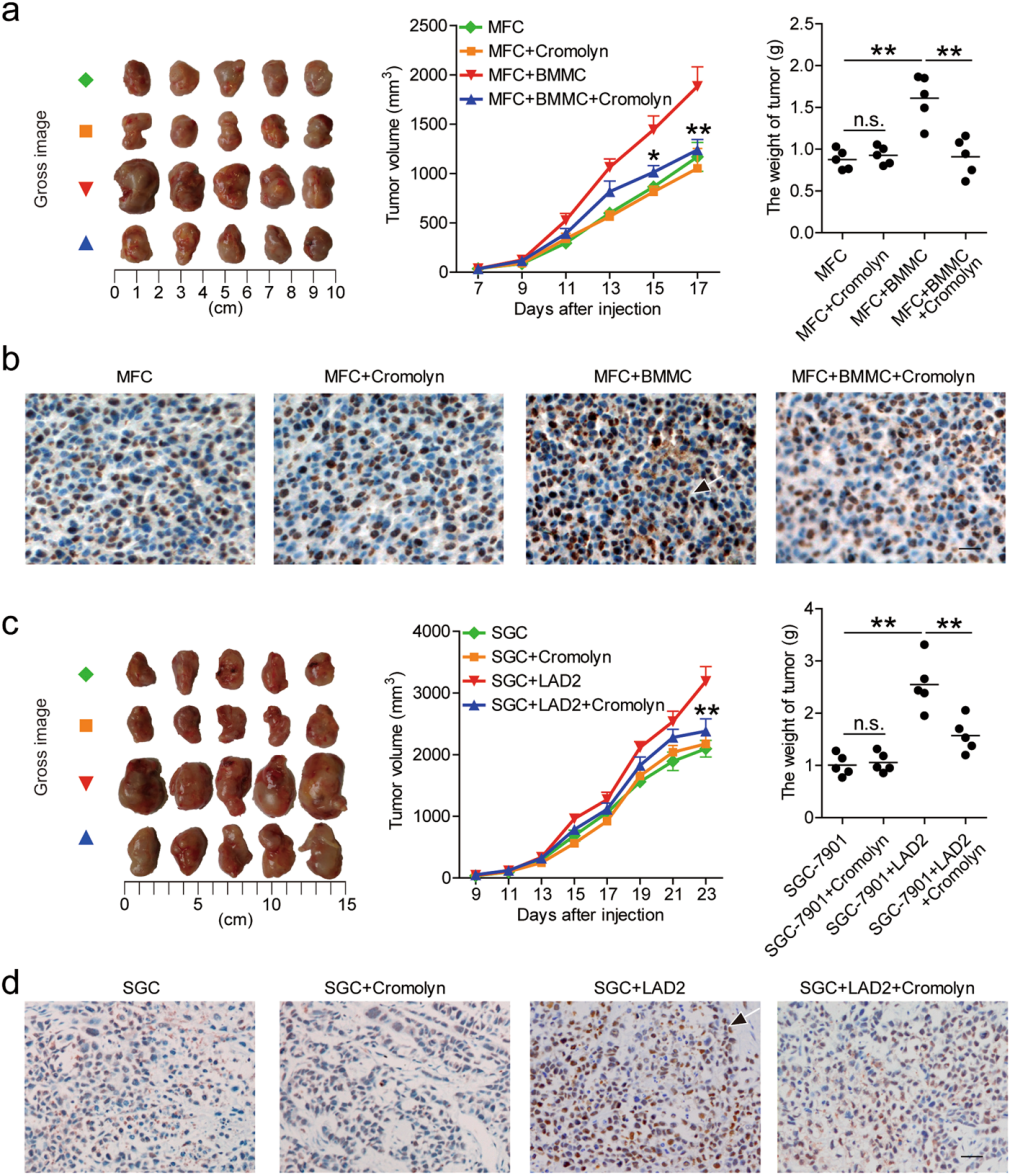
Mast cell-associated degranulation promotes GC progression in vivo. **a**, **c** Mice were injected with mouse MFC cells (**a**) or human SGC-7901 cells (**c**), as described in Materials and Methods. The control animals () received no further injections. The experimental treatments for mouse MFC cells-established tumors entailed injections with only Cromolyn () or BMMCs () or BMMCs plus Cromolyn (). The experimental treatments for human SGC-7901 cells-established tumors entailed injections with only Cromolyn () or LAD2 cells () or LAD2 cells plus Cromolyn (). The illustrated data represent tumor volumes (5 mice in each group). The day of tumor cell injection was counted as day 0. The weights of tumors were compared. Each dot in panel (**a**) and (**c**) represents 1 mouse. *P < 0.05, **P < 0.01, for groups injections with BMMCs or LAD2 cells (), compared with groups injections with BMMCs plus Cromolyn or LAD2 cells plus Cromolyn (). The tumors were excised and photographed 17 or 23 day after injecting the tumor cells. The proliferating cell nuclear antigen (PCNA) (brown) expression in tumors of mice was compared. Scale bars: 50 microns. Arrows indicate staining-positive cells. hCBMCs human umbilical cord blood-derived cultured mast cells, BMMCs bone marrow-derived mast cells, SGC SGC-7901, sup supernatant

To further see whether mast cell-associated degranulation operates in the promotion on tumor growth and GC progression in vivo, we injected BMMCs with or without mast cell degranulation inhibitor Cromolyn into our established NOD/SCID mice bearing mouse MFC-derived GC. Consistent with a vital role in assisting tumors of mast cell-associated degranulation in vivo, compared to mice injected with BMMCs, mice injected with BMMCs plus Cromolyn showed decreased tumor volumes, disease progression and tumor cell proliferation (Fig. 5a, b). Similar observations were made when using LAD2 cells in our established NOD/SCID mice bearing human SGC-7901-derived GC (Fig. 5c, d). These findings suggest that mast cell-associated degranulation promotes GC cell proliferation and suppresses its apoptosis in vitro and thereby contributes to tumor growth and GC progression in vivo.

### Mast cell-derived IL-17A contributes to tumor growth and GC progression

**Fig. 6:**
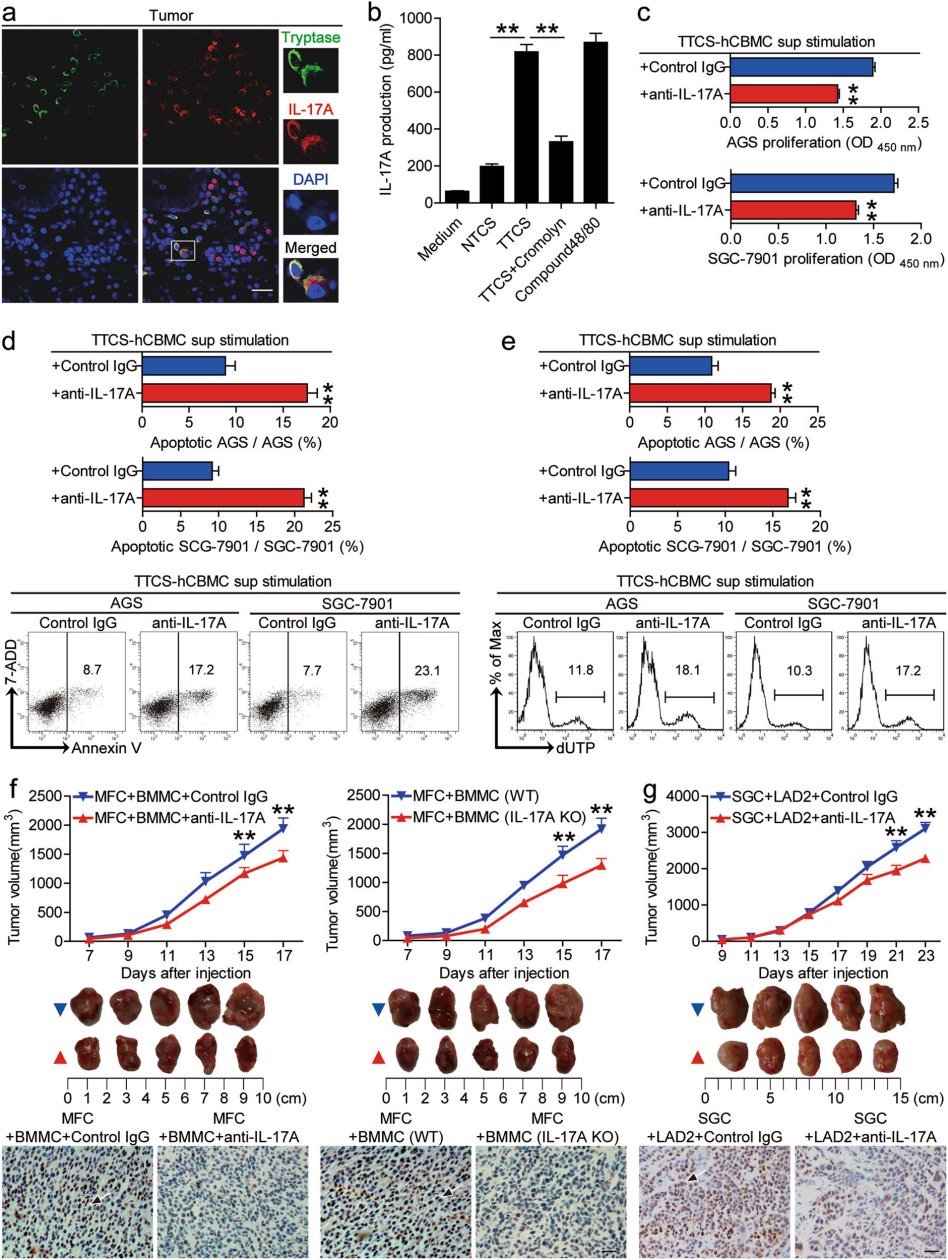
IL-17A derived from mast cells mediates tumor growth and GC progression. **a** Tumor-infiltrating IL-17A^+^tryptase^+^ mast cells were defined by immunofluorescence staining. Green, Tryptase; red, IL-17A; and blue, DAPI-stained nuclei. Scale bars: 20 microns. **b** The production of IL-17A from hCBMCs exposed to autologous NTCS, TTCS with or without Cromolyn was analyzed (*n* = 3). **P < 0.01, for groups connected by horizontal lines. **c**–**e** GC cells were stimulated with the culture supernatants from TTCS-conditioned hCBMCs (referred as TTCS-hCBMC) plus control IgG or IL-17A neutralizing antibodies, as described in Methods. The proliferation (**c**) of GC cells were analyzed (*n* = 3). The apoptosis of GC cells were analyzed by annexin V (**d**) and deoxyuridine triphosphate nucleotides (dUTP) (**e**) detection (*n* = 3). **P < 0.01, for groups stimulated with the culture supernatants from TTCS-hCBMCs plus IL-17A neutralizing antibodies, compared with groups stimulated with the culture supernatants from TTCS-hCBMCs plus control IgG. **f**, **g** Mice were injected with mouse MFC cells (**f**) or human SGC-7901 cells (**g**), as described in Methods. The control animals for mouse MFC cells-established or human SGC-7901 cells-established tumors received BMMCs plus control IgG or BMMCs from wild type (WT) mice or LAD2 cells (). The experimental treatments for mouse MFC cells-established or human SGC-7901 cells-established tumors entailed injections BMMCs plus IL-17A neutralizing antibodies or BMMCs from IL-17A-knockout (IL-17A KO) mice or LAD2 plus IL-17A neutralizing antibodies (). The illustrated data represent tumor volumes (5 mice in each group). The day of tumor cell injection was counted as day 0. **P < 0.01, for groups injections with mast cells plus IL-17A neutralizing antibodies or mast cells from IL-17A KO mice (), compared with groups injections with mast cells plus control IgG or mast cells from WT mice (). The tumors were excised and photographed 17 or 23 day after injecting the tumor cells. The proliferating cell nuclear antigen (PCNA) (brown) expression in tumors of mice was compared. Scale bars: 50 microns. Arrows indicate staining-positive cells. hCBMCs human umbilical cord blood-derived cultured mast cells, BMMCs bone marrow-derived mast cells, SGC SGC-7901, sup supernatant

Several biologically active factors could be secreted through degranulation from mast cells. To see which molecules from mast cells might have effects on GC cells, we first used some reported molecules (G-CSF, SCF, TGF-β, IL-3, IL-6, IL-17A, IL-22, IL-33) secreted by mast cells to stimulate GC cells, and found that only IL-17A remarkably induced GC cell proliferation (Supplementary Figure 4a). Besides, we found that ADM could remarkably induced IL-17A release from mast cells in a dose-dependent manner (Supplementary Figure 4b). Interestingly, mast cell-derived IL-17A could be blocked by inhibition of mast cell degranulation with Cromolyn (Fig. 6b). Next, to evaluate the potential role of mast cell-derived IL-17A on GC cells, we added neutralizing antibodies against IL-17A into the culture supernatants from TTCS-hCBMCs. Interestingly, blocking IL-17A efficiently inhibited GC cell proliferation (Fig. 6c) and promoted GC cell apoptosis assessed by annexin V (Fig. 6d) and dUTP (Fig. 6e) detection. Similar observations were made when using the culture supernatants from TTCS-conditioned LAD2 cells (referred as TTCS-LAD2) (Supplementary Figure 4c–e).

As for the expression of IL-17A in mast cells within GC tumors (Fig. 6a), and the high expression of IL-17A receptor A (IL-17RA) on GC cells (Supplementary Figure 4f), we blocked IL-17A with neutralizing antibodies or deleted IL-17A with IL-17A-knockout mast cells in our established NOD/SCID mice bearing mouse MFC-derived or human SGC-7901-derived GC. Consistent with a vital role in assisting tumors of mast cell-derived IL-17A in vivo, compared to mice injected with mast cells plus control IgG or mast cells from WT mice, mice injected with mast cells plus IL-17A neutralizing antibodies or mast cells from IL-17A knockout mice showed decreased tumor volumes, disease progression and tumor cell proliferation (Fig. 6f, g and Supplementary Figure 4g). These findings together suggest that mast cell-derived IL-17A promote GC cell proliferation and suppress GC cell apoptosis in vitro and thereby contribute to tumor growth and GC progression in vivo.

## Discussion

Illuminating the roles of host innate and adaptive immune cell within the tumor milieu is crucial for understanding the development and progression of human tumors^19^. Previous studies have been delineating the functions of adaptive immune cells in GC^5,20^, the roles of innate immune cells remain less well understood. Mast cells are a group of innate immune cells, which have been reported in GC^11,21^, but the presence of tumor-associated mast cells, as well as their precise mechanisms of communication in gastric cancer remains unclear. A better understanding of tumor and mast cell-derived factors modulating mechanisms such as mast cells degranulation, cancer cell growth, angiogenesis, or apoptosis resistance at the tumor body may prove to be rational biological targets for the therapeutic intervention of human cancers. Hence, we explore the hypothesis that gastric cancer-derived ADM induced mast cells activation and degranulation resulting in enhanced tumor promotion and progression.

In this study, we have shown that within GC mast cells play a positive role on promoting tumor progression. We have found that the infiltration number of mast cells in tumors was significantly increased at advanced stages of GC, with a high number of mast cells positively correlating with poor overall survival of GC patients. In addition, to our knowledge this is the first demonstration for tumor-derived ADM to induce mast cell degranulation in GC microenvironment, which exerts protumorigenic roles by facilitating tumor progression via releasing pro-inflammatory IL-17A.

In humans, mast cell infiltration in tumors influences disease progression and patient survival^22,23^. Hence, the analysis of mast cell infiltration in GC is a crucial area of clinical investigation. Our data shed some light on the clinical relevance of mast cells in GC. We observed a significant positive association between the number of mast cells infiltrated in GC and advanced clinical features of GC, such as tumor size (Fig. 1d). Besides, we found that an increased number of intratumoral mast cells predicted a lower rate of patient overall survival independently, as well as, disease-free survival was oppositely correlated with intratumoral mast cell levels (Fig. 1e, f), suggesting that tumor-infiltrating mast cells may become a helpful clinical prognostic marker in the future.

Mast cells are long-lived secretory cells, able to rapidly respond to modifications in their microenvironment and release large amounts of preformed and pre-activated immunomodulatory compounds through degranulation^24,25^. The well-known mechanism of mast cell activation is the engagement of the high-affinity receptor for IgE immunoglobulins in anaphylactic reactions^7^, however, the mechanisms of mast cell degranulation were not well understood in GC. In view of this, we focused on the potential mechanism and found an IgE-independent manner of mast cell activation in GC. In our study, we detected β-hexosaminidase release as an indicator of mast cell degranulation, and found that TTCS could activate and induce mast cell degranulation. It is known that the granule release by mast cells degranulation would result in a massive proinflammatory response at the tumor site that may be detrimental for cell survival. Alternatively, selective release of critical granules containing trophic or mitogenic factors would establish an enriching microenvironment to drive tumor proliferation. So, we screened pro-inflammatory molecules in human GC microenvironments by microarray (Fig. 3a), and stimulated mast cells with highly-expressed molecules. We found that only ADM remarkably induced mast cell degranulation in a dose-dependent manner (Fig. 4b, c). ADM is a 52-amino acid peptide amide which has multifunction and participates in human carcinogenesis through diverse mechanisms^15^. In many human malignancies, the level of ADM is often elevated, including cancers of the brain, lung, colon and others^26^. In our study, we identified an increased ADM within GC microenvironment, meanwhile, the tumor-derived ADM effectively induced mast cell degranulation by activating PI3K-ATK pathway. Besides, some studies revealed the presence of ADM-producing mast cells in intratumoral tissue by immunohistochemical analysis from patients with lung or breast cancer^26,27^. This clinical finding further implicates ADM-mediated autocrine/paracrine interactions between tumor and mast cells, which is a potent angiogenic factor with similar molar activity to VEGF and bFGF.

Tumor-associated mast cells can also be activated to secrete an amount of biological molecules to mediate tumor progression^8,28^. Previous studies had found that mast cell released histamine via c-Kit/SCF, which increases cholangiocarcinoma growth, and angiogenesis^29^. Moreover, mast-cell-derived mediators: histamine, CXCL1 and CXCL10 could induce thyroid cancer cell survival and DNA synthesis in vitro^30^. In pancreatic cancer, activated mast cells promote tumor progression by IL-13 and tryptase^22^. IL-17A is a pleiotropic proinflammatory cytokine, participating in regulating tumor progress^31,32^ and associating with prognosis of carcinoma^33^. We demonstrated that mast cell-derived IL-17A can promote the proliferation and inhibit the apoptosis of GC cells in vitro. Importantly, with in vivo GC models, our hypothesis that mast cells effectively produce IL-17A by degranulation to facilitate GC progression was fully verified. In line with our data, a recent paper showed the effect of promoting growth of B cell non-Hodgkin lymphomas depended on IL-17A overexpression in NOD/SCID mice^34^. Recently, some new mechanisms of tumor proliferation have been found in digestive system neoplasm. Makino Y showed that hepatocellular carcinoma (HCC) derived connective tissue growth factor (CTGF) could mediates tumor-stroma interactions between hepatoma cells and hepatic stellate cells to accelerate HCC progression^35^. In colorectal tumor microenvironment, histidine decarboxylase (HDC)^+^ myeloid cells-derived CXCL13/CXCR5 axis that mediated Foxp3 expression and Treg proliferation, which affected CD8^+^ T cells directly and thus appeared to play key roles in suppressing tumoricidal immunity and facilitated colorectal tumor proliferation^36^. In our study, we further demonstrated that such tumor growth was through IL-17A from mast cells as blocking IL-17A reversed such growth. It is highly likely that in different tumors tumor-infiltrating mast cells may play their protumorigenic roles via different bioactive molecules.

**Fig. 7:**
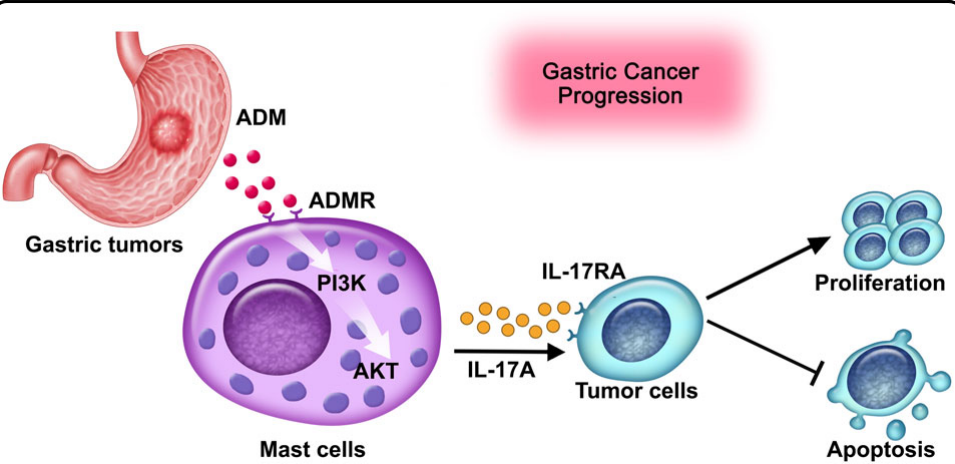
A proposed model of cross-talks among tumor cells, ADM, mast cells, and IL-17A leading to mast cell-mediated protumorigenic effects in the GC microenvironment. Tumor-derived ADM induces mast cell degranulation via PI3K-AKT signaling pathway activation. These activated mast cells exert a protumorigenic effect by releasing IL-17A, which promotes GC progression

Collectively, based on our in vitro and in vivo data, we propose a model involving complex interactions between ADM, mast cells, IL-17A and tumor cells within GC (Fig. 7). First, increased tumor-derived ADM induces mast cell degranulation via PI3K-AKT signaling pathway. Next, these activated mast cells exert a protumorigenic effect through releasing IL-17A, which promotes GC progression. In conclusion, our study has highlighted a notable role for mast cells in human GC and identified new mechanisms of GC–associated mast cells mediating tumor progression via secreting proinflammatory factor-dependent manners. Overall, blocking the function of mast cells that infiltrate tumors or the secreting protumorigenic mediators from mast cells may be a useful therapeutic strategy for preventing GC progress.

## Materials and Methods

### Patients and specimens

Fresh gastric tumor (homogeneous cellularity, without foci of necrosis, including intratumoral and marginal tissues), peritumoral and non-tumor (non-tumor tissues, at least 5 cm distant from the tumor site) tissues and autologous peripheral blood were obtained from patients with GC who underwent surgical resection at the Southwest Hospital of Third Military Medical University. Before taking the samples, none of these patients had received chemotherapy or radiotherapy. Patients with infectious diseases, autoimmune diseases, or multi-primary cancers were excluded. The stages of tumors were determined according to the TNM classification system of the International Union Against Cancer (7th edition). The study was approved by the Ethics Committee of the Southwest Hospital of Third Military Medical University. Written informed consent was obtained from each subject. Antibodies and other reagents were listed in Supplementary Table 1.

### Preparation of TTCS and NTCS

Tumor tissue culture supernatants (TTCS) or non-tumor tissue culture supernatants (NTCS) were prepared by plating autologous tumor or non-tumor gastric tissues (0.5 cm^3^) in 1 ml RPMI 1640 medium for 24 h. The supernatant was then centrifuged and harvested. RPMI 1640 was referred as Medium, which was set as a non-conditioned media control.

### Preparation of the culture supernatants of TTCS-conditioned or NTCS-conditioned mast cells

TTCS or NTCS were prepared as above. hCBMCs or LAD2 cells were cultured with autologous 50% TTCS or NTCS for 24 h. Then the culture supernatants were centrifuged and harvested. TTCS-conditioned or NTCS-conditioned mast cells were referred as TTCS- or NTCS-mast cells in the following text.

### Mast cell degranulation measured by β-hexosaminidase release assay

Analysis of tumor microenvironment-induced β-hexosaminidase release from human mast cells was accomplished by following previously established protocol^26^. Briefly, hCBMCs or LAD2 cells (1 × 10^5^ cells/well in 96-well plates) were suspended in Tyrodes buffer (10 nmol/L HEPES, 137 mmol/L NaCl, 2.7 mmol/L KCl, 0.4 mmol/L Na_2_HPO_4_, 5.6 mmol/L D-glucose, 1.8 mmol/L CaCl_2_, 1.3 mmol/L MgSO_4_) containing 0.025% bovine serum albumin (BSA). Autologous 50% TTCS (with or without ADM Fragment 22–52 (AMA) (1 μM) or Cromolyn (250 μM)) or NTCS, or appropriate concentrations of ADM (10, 50, 100 ng/ml) or different cytokines (GM-CSF, M-CSF, G-CSF, IFN-γ, TGF-β, TNF-α, IL-1β, IL-6, IL-10, IL-17A, IL-22) (100 ng/ml) were added into mast cell suspensions and incubated for 60 minutes at 37 °C to stimulate β- hexosaminidase release. The release of β-hexosaminidase in the supernatants and the cell pellets (solubilizing with 0.5% Triton X-100 in Tyrode’s buffer) were measured with p-nitrophenyl N-acetyl-b-D-glucosaminide (PNAG) in 0.1 M sodium citrate (pH = 4.5) for 60 minutes at 37 °C. The reaction was stopped using 0.4 mol/L glycine (pH = 10.7) and the absorbance was determined at 405 nm. Resulting beta-hexosaminidase activity was calculated as (mean ± SEM): [(β-hexosaminidase in supernatant × 2)/(β-hexosaminidase in supernatant + {total β-hexosaminidase in cell pellet × 4})] × 100%. RPMI 1640 medium and Compound48/80 (5 μg/ml) were placed in the cell cultures as blank and positive controls respectively.

Besides, for the signaling pathway inhibition experiments, LAD2 cells were pretreated with 2 μl AG490 (a JAK inhibitor), BAY 11-7082 (an IκBα inhibitor), SP600125 (a c-Jun N-terminal kinase (JNK) inhibitor), SB203580 (a mitogen-activated protein kinase (MAPK) inhibitor), U0126 (MEK-1 and MEK-2 inhibitor), or Wortmannin (a PI3K inhibitor) (10 μM) for 2 h, then the cells were stimulated with 50% TTCS for 1 h and harvested as above. Since the inhibitors were dissolved in DMSO, parallel cell groups were pretreated with DMSO (2 μl) or culture media as controls. Then the β-hexosaminidase release was measured as above.

### GC cell proliferation and apoptosis assay

Human GC cell lines AGS and SGC-7901 were stimulated with - 50% TTCS or NTCS, the culture supernatants of hCBMCs or LAD2 cells, the culture supernatants of TTCS-conditioned or NTCS-conditioned hCBMCs or LAD2 cells, or different cytokines (G-CSF, SCF, IFN-γ, TGF-β, IL-3, IL-6, IL-17A, IL-22, IL-33) (100 ng/ml) for 72 h, the cell proliferation was measured by CCK-8 (Dojindo) and the cell apoptosis was quantified using Annexin V Apoptosis Detection Kit I (BD Biosciences) or APO-Direct Apoptosis Detection (eBioscience) according to the manufacturer’s instructions. In some cases, human IL-17A neutralizing antibody (20 μg/ml) or control IgG (20 μg/ml) was added in the culture system.

### In vivo tumor inhibition assay

All animal experiments were undertaken with the approval from the Animal Ethical and Experimental Committee of Third Military Medical University. 10^6^ GC cells (MFC or SGC-7901 cells) in 100 μl of buffered saline were subcutaneously injected into the axillary tissues of female nonobese diabetic/severe combined immunodeficiency (NOD/SCID) mice (5–7 week, one tumor per mouse). Once the xenografts of GC reached the volume of 40 mm^3^, Cromolyn (10 mg/kg) dissolved in saline was administered intraperitoneally daily (the control group injected with saline). The next day after the first Cromolyn injection, 5 × 10^5^ mast cells (BMMCs from WT or IL-17A knockout mice or LAD2 cells) in 50 μl of buffered saline were injected into the tumor cavity (the control group injected with saline). In some cases, neutralizing antibodies against human/mouse IL-17A (20 μg per mouse) or control IgG (20 μg per mouse) were subsequently injected into the peritoneum in 100 μl of buffered saline every 2 days after the mast cell injection. Tumor size was measured every 2 days by two independent observers using calipers fitted with a vernier scale. Tumor volumes (*V*) were calculated with the formula: *V* = *A* × *B*^2^/2 (*A* = axial diameter; *B* = rotational diameter). Once the mice were killed, tumors were weighed and photographed, and were further fixed for immunohistochemical staining.

### Statistical analysis

Results are expressed as mean ± SEM. Student *t* test was generally used to analyze the differences between two groups, but when the variances differed, the Mann–Whitney *U* test was used. For multigroup data analysis, an ANOVA analysis was used. Correlations between parameters were assessed using the Pearson correlation analysis and linear regression analysis as appropriate. Overall/disease-free survival was defined as the interval between surgery and death/recurrence or between surgery and the last observation for surviving/disease-free patients. The known tumor-unrelated deaths (eg, accidental death) were excluded from the death record for this study. Cumulative survival time was calculated by the Kaplan-Meier method, and survival was measured in month; the log-rank test was applied to compare between 2 groups. Multivariate analysis of prognostic factors for patient overall survival was performed using the Cox proportional hazards model. SPSS statistical software (version 13.0) was used for all statistical analysis. All data were analyzed using 2-tailed tests, and *P* < 0.05 was considered statistically significant.

## Electronic supplementary material

Supplementary Figures Legends

Supplementary Methods

Supplementary Table 1

Supplementary Figure 1

Supplementary Figure 2

Supplementary Figure 3

Supplementary Figure 4
