# Supplementary material for: Degranulation of mast cells induced by gastric cancer-derived adrenomedullin prompts gastric cancer progression
Source: Cell Death Dis. 2018 Oct 10;9(10):1034. doi: 10.1038/s41419-018-1100-1 (PMC6180028; doi:10.1038/s41419-018-1100-1)
Supplement: Supplementary file 1 — Supplementary Figures Legends [file 41419_2018_1100_MOESM1_ESM.doc]

**Supplementary Figure Legends**

**Supplementary Figure 1.** Identification of human umbilical cord blood-derived cultured mast cells (hCBMCs) and correlations of mast cell number in GC with clinical parameters. (**a**) Surface staining of FcεRI and CD117 of generated human umbilical cord blood-derived cultured mast cells (hCBMCs) was shown. Results were expressed as percentage of CD117^+^FcεRI^+^ cells by gating on CD45^+^ cells. Iso, Isotype control antibody. (**b**) Cellular morphology of hCBMCs was shown by light microscope. (**c**) Toluidine blue staining of hCBMCs was shown. Scale bars: 10 microns. (**d**) Mast cell number per field and its potential correlations with clinical parameters. **, *P*<0.01, n.s., *P*>0.05 for groups connected by horizontal lines. Each dot represents one patient. CEA, carcinoembryonic antigen; *H.pylori* Ab, *Helicobacter pylori* antibody.

**Supplementary Figure 2.** Tumor-derived ADM induces mast cell degranulation. (**a**) Mast cell degranulation and ADM concentration between autologous tumor and non-tumor tissues (n=65) was analyzed. The correlations between mast cell degranulation and ADM concentration in GC tumors (n=63) were analyzed. (**b**) Representative analysis of ADM-expressing (red) EpCam^+^ tumor cells (green) in tumor tissues of GC patients by immunofluorescence. Scale bars: 50 microns. (**c**) ADM production between autologous tumor tissue culture supernatants (TTCS) and non-tumor tissue culture supernatants (NTCS) (8 pairs) were analyzed by ELISA. Each dot in panel **a** represents 1 patient. *, *P*<0.05, **, *P*<0.01 for groups connected by horizontal lines.

**Supplementary Figure 3.** Mast cell-associated degranulation promotes tumor growth and GC progression. (**a**-**c**) GC cells were stimulated with different culture supernatants, as described in Methods. The proliferation (**a**) of GC cells were analyzed (n=3). The apoptosis of GC cells were analyzed by annexin V (**b**) and deoxyuridine triphosphate nucleotides (dUTP) (**c**) detection (n=3). *, *P*<0.05, **, *P*<0.01, n.s., *P*>0.05 for groups connected by horizontal lines. sup, supernatant.

**Supplementary Figure 4.** Mast cell-derived IL-17A promotes tumor growth and GC progression. (**a**) The proliferation of GC cells exposed to IL-17A, G-CSF, SCF, TGF-β, IL-3, IL-6, IL-22, IL-33 was analyzed (n=3). (**b**) The IL-17A release of hCBMCs stimulated by ADM was analyzed (n=3). (**c**-**e**) GC cells were stimulated with the culture supernatants from TTCS-conditioned LAD2 cells (referred as TTCS-LAD2) plus control IgG or IL-17A neutralizing antibodies, as described in Methods. The proliferation (**c**) of GC cells were analyzed (n=3). The apoptosis of GC cells were analyzed by annexin V (**d**) and deoxyuridine triphosphate nucleotides (dUTP) (**e**) detection (n=3). (**f**) Expression of IL-17A receptor A (IL-17RA) on tumor-infiltrating GC tumor cells, AGS cells or SGC-7901 cells. black, isotype control. (**g**) Mice were injected with mouse MFC cells with BMMCs plus control IgG or IL-17A neutralizing antibodies or BMMCs from wild type (WT) or IL-17A-knockout (IL-17A KO) mice, or injected with human SGC-7901 cells with LAD2 cells plus control IgG or IL-17A neutralizing antibodies, as described in Methods. The weights of tumors were compared. The horizontal bars in panel **g** represent mean values. Each dot in panel **g** represents 1 mouse. *, *P*<0.05, **, *P*<0.01 for groups connected by horizontal lines. SGC, SGC-7901; sup, supernatant.
