# Supplementary material for: Degranulation of mast cells induced by gastric cancer-derived adrenomedullin prompts gastric cancer progression
Source: Cell Death Dis. 2018 Oct 10;9(10):1034. doi: 10.1038/s41419-018-1100-1 (PMC6180028; doi:10.1038/s41419-018-1100-1)
Supplement: Supplementary file 2 — Supplementary Methods [file 41419_2018_1100_MOESM2_ESM.doc]

**Supplementary Materials and Methods**

**Mast cell and** **GC cell culture**

For culture of primary human umbilical cord blood-derived cultured mast cells (hCBMCs), human umbilical cord blood was obtained from obstetrics and gynecology department in the Southwest Hospital of Third Military Medical University. Umbilical cord blood mononuclear cells were isolated by density gradient centrifugation using Ficoll-Paque Plus. CD133^+^ cells were purified with CD133 microbeads. For the first 6 weeks, CD133^+^ cells were cultured in StemSpan Serum-Free Expansion Medium supplemented with penicillin (100 U/ml)/streptomycin (100 µg/ml), human recombinant (hr) stem cell factor (SCF) (100 ng/ml), hr IL-6 (50 ng/ml), hr IL-3 (30 ng/ml, only presents during the first 3 weeks). From week 6, fetal calf serum (FCS) (10%) was added into the culture medium. The generated mast cells were used for experiments until week 10. Mast cell purity was determined by surface staining of FcεRI and CD117, and toluidine blue staining.

The human mast cell line LAD2 (kindly provided by Dr. Wei Zhang, Shenzhen Key Laboratory for Translational Medicine of Dermatology, China) were cultured in Serum-Free Media (StemPro-34) supplemented with penicillin (100 U/ml)/streptomycin (100 µg/ml), hr SCF (100 ng/ml), hr IL-6 (50 ng/ml) and L-glutamine (2 mM) as described previously ^1^.

Primary murine bone marrow-derived mast cells (BMMCs) were isolated from the femoral lavage of wild-type (WT) C57BL/6 mice or IL-17A knockout (IL-17A KO) mice (C57BL/6 background, kindly provided by Prof. Richard A. Flavell, Yale University School of Medicine) and cultured in complete RPMI 1640 medium supplemented with 10% FCS (R-10) in the presence of mouse recombinant (mr) IL-3 (10 ng/ml), mr SCF (10 ng/ml) and L-glutamine (2 mM). The non-adherent cells were passaged every 3 days. 4 weeks later, the generated mast cells were verified by surface staining of FcεRI, CD117 and toluidine blue staining before being used for experiments.

Human GC cell lines AGS, SGC-7901 and mouse GC cell line MFC were purchased from China Center for Type Culture Collection (CCTCC, Wuhan, China) and cultured according to their guidelines.

**Western blot**

Western blot assays were performed on 10%-15% SDS-PAGE gels using equivalent amounts of cell lysate proteins of samples. 5% BSA was used for blocking the PVDF membranes. Human AKT, p-AKT (ser473) and ADM were detected with rabbit anti-AKT, rabbit anti-p-AKT (ser473) and rabbit anti-ADM antibodies respectively. This was followed by incubation with horseradish peroxidase (HRP)-conjugated secondary antibodies. Bound proteins were visualized by using SuperSignal® West Dura Extended Duration Substrate kit.

**ELISA**

Human gastric tissues were collected, homogenized in 1 ml sterile Protein Extraction Reagent, and centrifuged. Tissue supernatants were collected for ELISA. Cell culture supernatants were collected as above for ELISA. Concentrations of β-hexosaminidase and ADM in the tumor or non-tumor gastric tissues and TTCS or NTCS; and concentrations of IL-17A in the mast cell culture supernatants were determined using ELISA kits according to the manufacturer’s instructions.

**Immunohistochemistry**

Paraformaldehyde-fixed and paraffin-embedded samples were cut into 5 µm sections. For immunohistochemical single-staining, the sections were incubated with mouse anti-human/mouse proliferating cell nuclear antigen (PCNA), or mouse anti-human tryptase antibodies respectively, either followed by HRP-conjugated anti-mouse IgG or the EnVision G2 System/AP Rabbit/Mouse (Permanent Red) followed by diaminobenzidine. All the sections were finally counterstained with haematoxylin and examined using a microscope (Nikon Eclipse 80i; Nikon).

**Immunofluorescence**

Paraformaldehyde-fixed tumor tissue sections from GC patients were washed in PBS, blocked for 30 min with 20% goat serum in PBS, then stained for RAMP2 and tryptase, or ADM and EpCam. Slides were examined with a confocal fluorescence microscope (LSM 510 META, Zeiss).

**Real-time PCR**

RNA of gastric tumor and non-tumor tissues was extracted with TRIzol reagent. The RNA samples were reverse transcribed to cDNA with PrimeScriptTM RT reagent Kit. Real-time PCR was performed on the IQ5 (Bio-Rad) with the Real-time PCR Master Mix according to the manufacturer's specifications. Expression of ADM was measured using the SYBR green method with primers (forward: 5’-TTGGATGTCGCGTCGGAG-3’, reverse: 5’-TAGCTGCTGGACATCCGCA-3’). Human GAPDH served as the normaliser. The relative gene expression was expressed as fold change calculated by the ΔΔCt method.

**Microarray experiments**

Gene expression profiles of human tumor tissues from GC patients were analyzed with the Affymetrix GeneChip Human Gene 1.0 ST Array (Affymetrix), strictly following the manufacturer’s protocol. Microarray experiments were performed at the Genminix Informatics (China) with the microarray service certified by Affymetrix.

**References:**

1. Radinger M, Jensen BM, Kuehn HS, Kirshenbaum A, Gilfillan AM. Generation, isolation, and maintenance of human mast cells and mast cell lines derived from peripheral blood or cord blood. *Curr Protoc Immunol* 2010; **Chapter 7**:7-37.
