# Supplementary material for: Degranulation of mast cells induced by gastric cancer-derived adrenomedullin prompts gastric cancer progression
Source: Cell Death Dis. 2018 Oct 10;9(10):1034. doi: 10.1038/s41419-018-1100-1 (PMC6180028; doi:10.1038/s41419-018-1100-1)
Supplement: Supplementary file 3 — Supplementary Table 1 [file 41419_2018_1100_MOESM3_ESM.doc]

**Supplementary Table 1.** Antibodies and other reagents

| Antibodies and reagents | Manufacturers |  |
| --- | --- | --- |
| Antibodies for immunohistochemical staining |  |  |
| anti-human tryptase | Abcam |  |
| anti-human proliferating cell nuclear antigen (PCNA) | Santa Cruz |  |
| horseradish peroxidase anti-rabbit IgG  horseradish peroxidase anti-mouse IgG  EnVision™ G2 System/AP Rabbit/Mouse (Permanent Red) | Zhongshan Biotechnology  Zhongshan Biotechnology  Dako |  |
| Antibodies for immunofluorescence  mouse anti-human tryptase  mouse anti-human EpCam  rabbit anti-human ADM  rabbit anti-human PAMP2  rabbit anti-human IL-17A  goat anti-rabbit-TRITC  goat anti-mouse-FITC | Abcam  Abcam  Abcam  Invitrogen  Abcam  Zhongshan Biotechnology  Zhongshan Biotechnology |  |
| Antibodies for neutralizing and blocking |  |  |
| anti-human IL-17A (Mouse IgG1 kappa) | eBioscience |  |
| anti-mouse IL-17A (Mouse IgG1 kappa)  Mouse IgG1 kappa Isotype Control | eBioscience  eBioscience |  |
| Antibodies for western blot |  |  |
| anti-human ADM | Abcam | |
| anti-human AKT | Cell signaling technology |  |
| anti-human p-AKT (ser473)  anti-human GAPDH | Cell signaling technology  Beijing Ray Antibody Biotech |  |
| ELISA kits |  |  |
| β-hexosaminidase  ADM | CUSABIO  CUSABIO | |
| IL-17A | Biolegend |  |
| Reagents for signaling pathways inhibition |  |  |
| MEK-1 and MEK-2 inhibitor U0126 | Merk Millipore |  |
| JAK signaling inhibitor AG490 | Merk Millipore |  |
| IκBα inhibitor BAY 11-7082 | Calbiochem |  |
| JNK inhibitor SP600125 | Calbiochem |  |
| MAPK inhibitor SB203580 | Calbiochem |  |
| PI3K inhibitor Wortmannin | Calbiochem |  |
| CD133 microbeads | Milteniy Biotec |  |
| Annexin V Apoptosis Detection Kit I | BD Biosciences |  |
| APO-Direct Apoptosis Detection | Invitrogen |  |
| CCK-8 Kits | Dojindo |  |
| ADM Fragment 22-52 (AMA) | Sigma-Aldrich |  |
| ADM | Sigma-Aldrich |  |
| Compound48/80 | Sigma-Aldrich |  |
| p-nitrophenyl N-acetyl-b-D-glucosaminide (PNAG) | Sigma-Aldrich |  |
| glycine | Sangon Biotech |  |
| DMSO | Sigma-Aldrich |  |
| Cromolyn | Sigma-Aldrich |  |
| Protein Extraction Reagent | Pierce |  |
| SuperSignal® West Dura Extended Duration Substrate kit | Thermo |  |
| Fetal calf serum (FCS) | Gibco |  |
| Penicillin/Streptomycin | Gibco |  |
| RPMI-1640 | Hyclone |  |
| StemSpan Serum-Free Expansion Medium  Serum-Free Media (StemPro-34) | StemCell Technologies  Life Technologies |  |
| L-glutamine | Gibco |  |
| Ficoll-Paque Plus | GE Healthcare |  |
| lyses solution  TRIzol reagent | TIANGEN  Invitrogen |  |
| PrimeScriptTM RT reagent Kit | TaKaRa |  |
| Real-time PCR Master Mix | Toyobo |  |
| All recombinant human/mouse cytokines and chemokines | PeproTech |  |

APC-Cy7, allophycocyanin-cyanin 7; PE-Cy7, phycoerythrin-cyanin 7; FITC, Fluorescein isothiocyanate; PE, phycoerythrin; PerCP-Cy5.5, peridin chlorophyl protein-cyanin 5.5; APC, allophycocyanin; IL, interleukin; I IFN, interferon; PD-L1, programmed death-ligand 1; ADM, adrenomedullin; EpCam, epithelial cell adhesion molecule; PCNA, proliferating cell nuclear antigen; RAMP2, receptor activity modifying protein 2.
