# Supplementary figures and images for: Degranulation of mast cells induced by gastric cancer-derived adrenomedullin prompts gastric cancer progression
Source: Cell Death Dis. 2018 Oct 10;9(10):1034. doi: 10.1038/s41419-018-1100-1 (PMC6180028; doi:10.1038/s41419-018-1100-1)

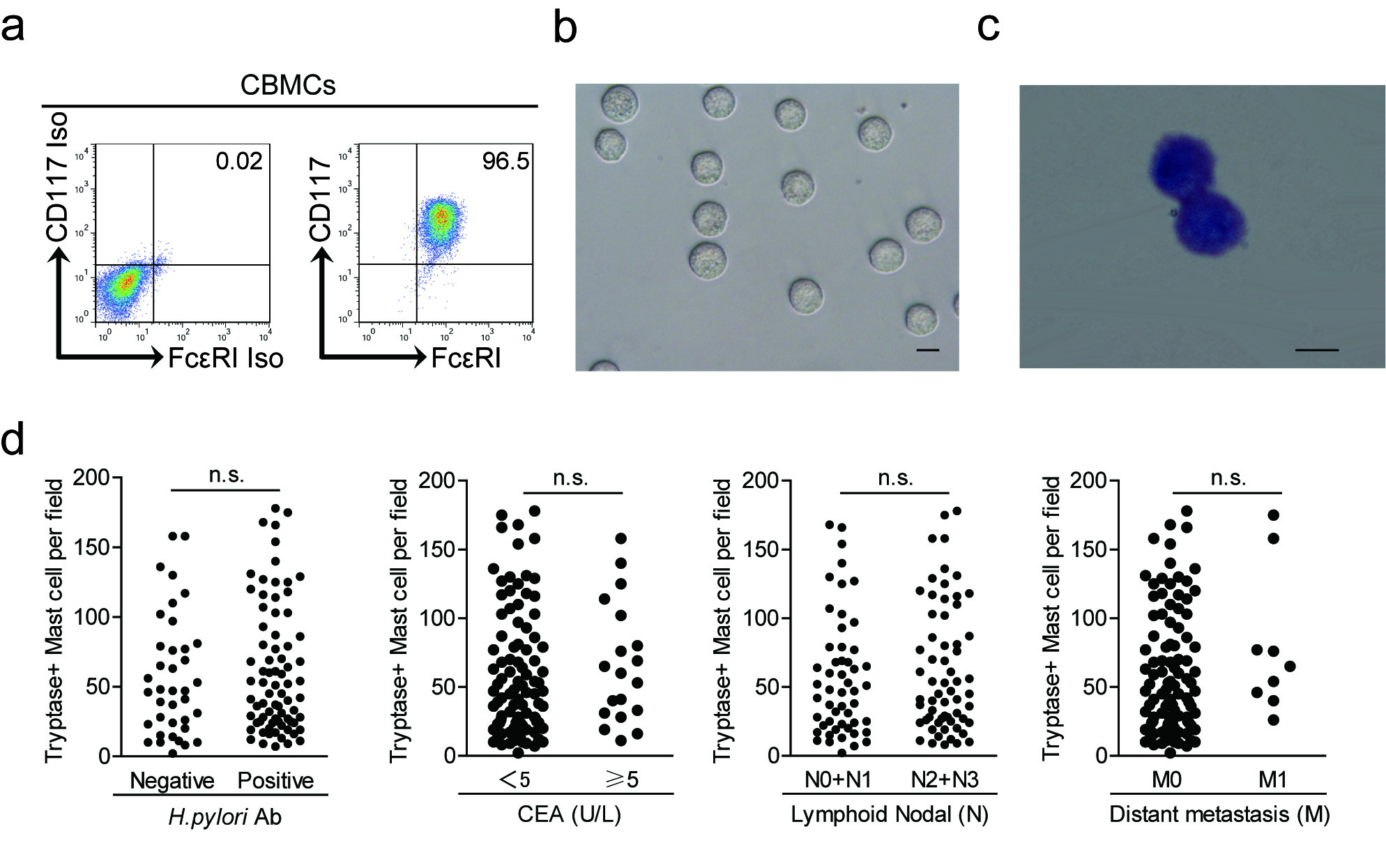

Supplement: Supplementary file 4 — Supplementary Figure 1 [file 41419_2018_1100_MOESM4_ESM.jpg]

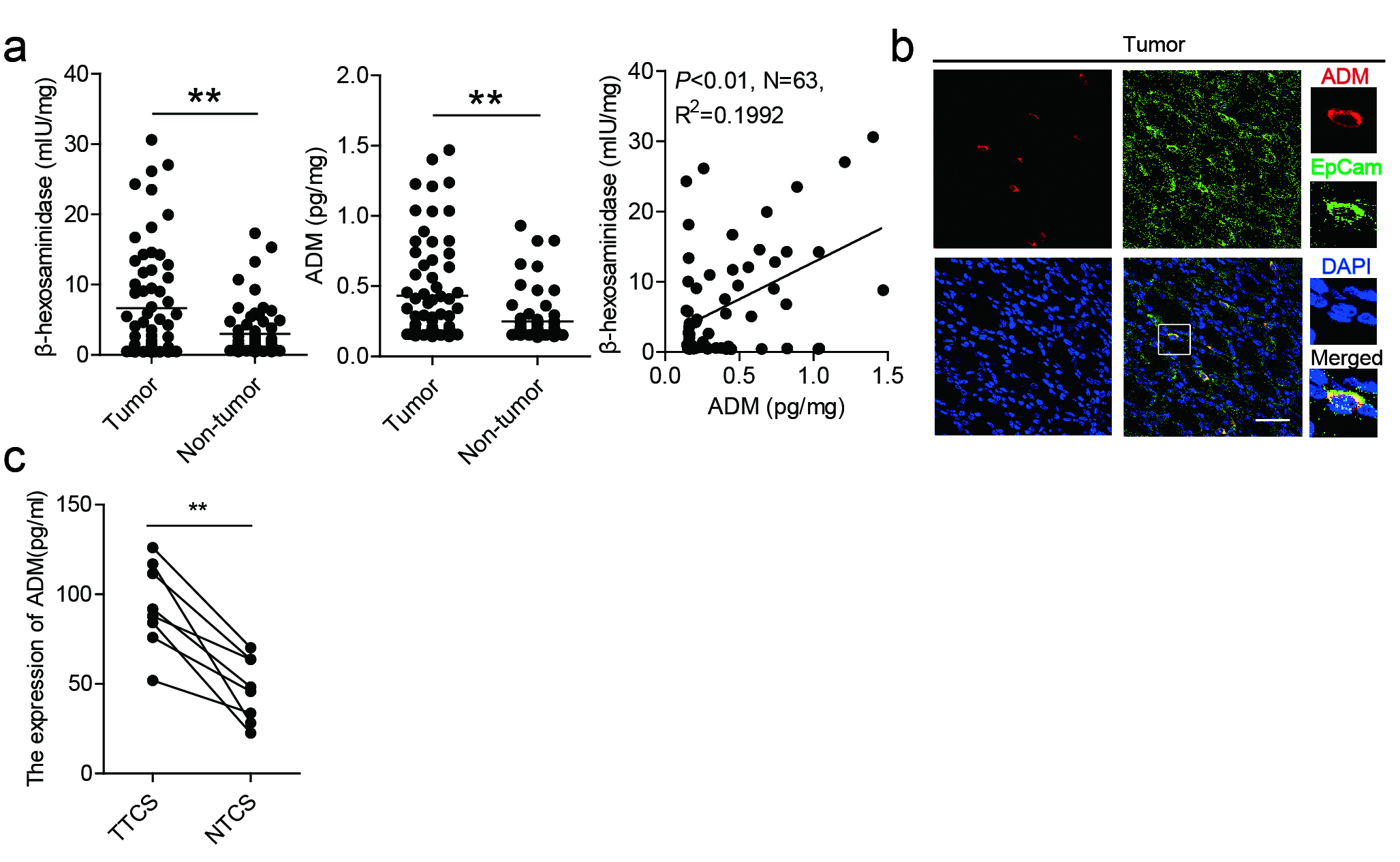

Supplement: Supplementary file 5 — Supplementary Figure 2 [file 41419_2018_1100_MOESM5_ESM.jpg]

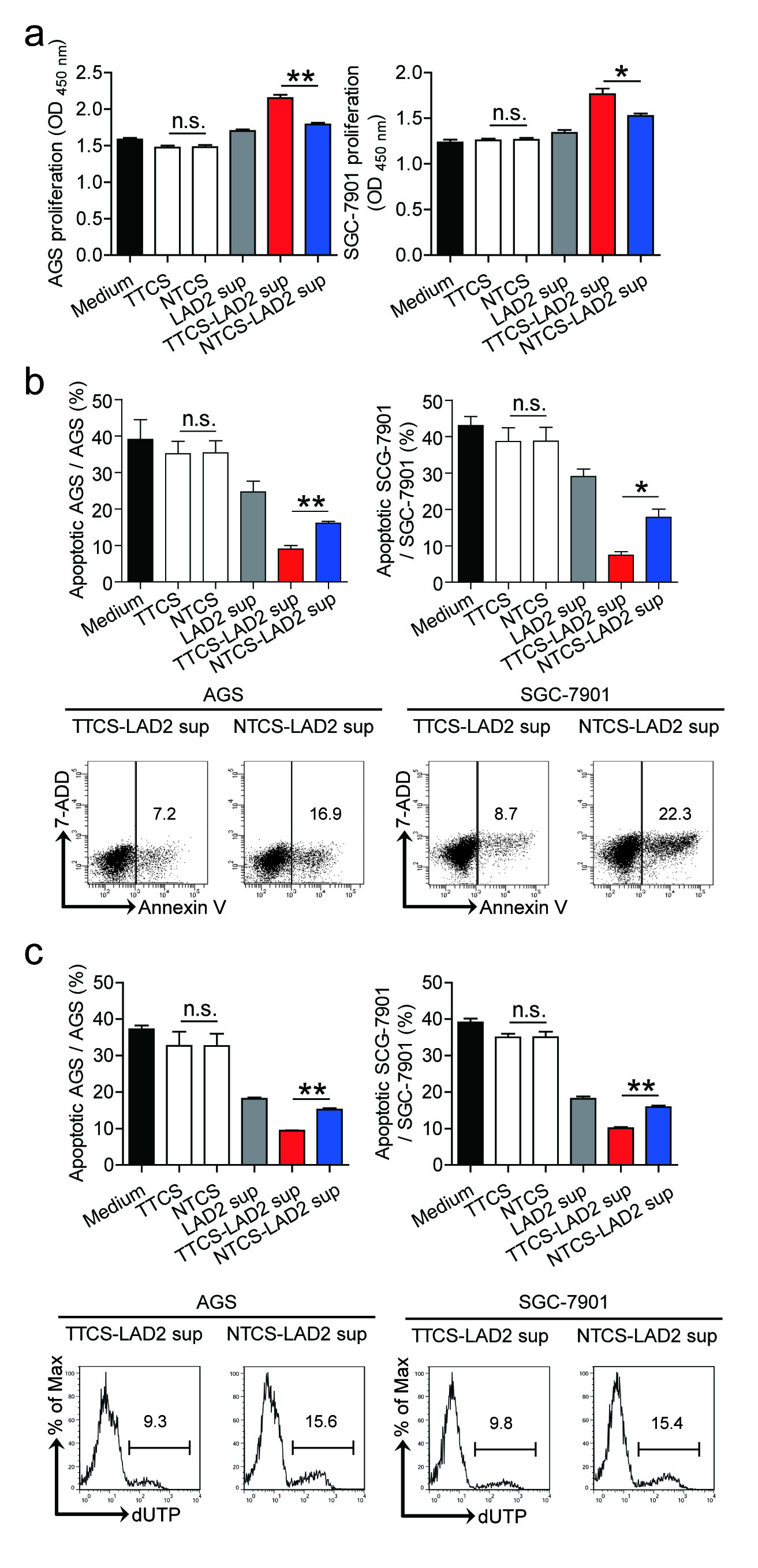

Supplement: Supplementary file 6 — Supplementary Figure 3 [file 41419_2018_1100_MOESM6_ESM.jpg]

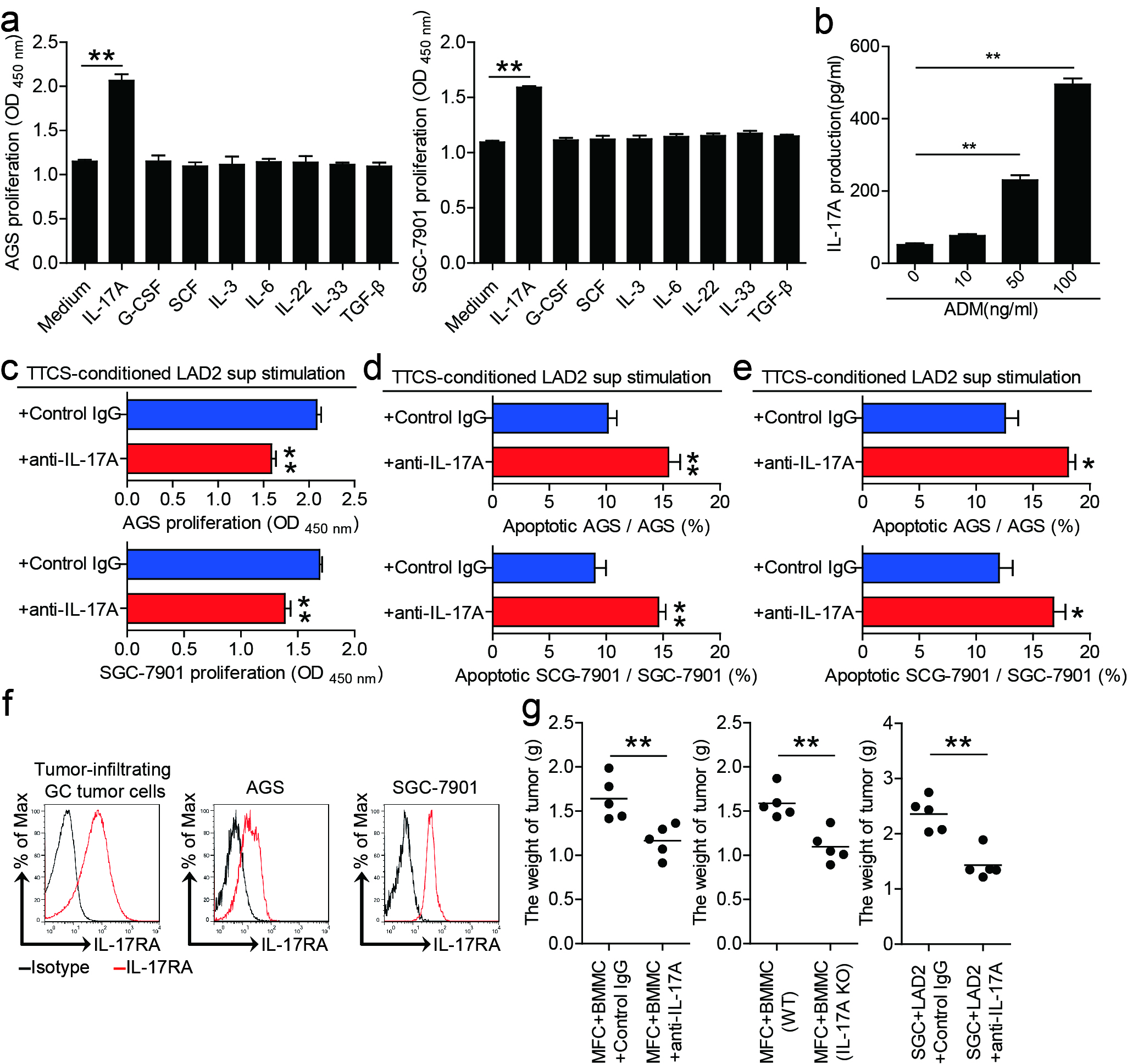

Supplement: Supplementary file 7 — Supplementary Figure 4 [file 41419_2018_1100_MOESM7_ESM.jpg]
